# Significant Osseous Metaplasia in a Chondroid Syringoma: A Case Report

**DOI:** 10.7759/cureus.72690

**Published:** 2024-10-30

**Authors:** Shristi Butta, Shivam Chakraborty, Keya Basu, Ayesha Islam

**Affiliations:** 1 Oncopathology, Institute of Post-Graduate Medical Education and Research (IPGMER) and Seth Sukhlal Karnani Memorial (SSKM) Hospital, Kolkata, IND; 2 Pathology, Institute of Post-Graduate Medical Education and Research (IPGMER) and Seth Sukhlal Karnani Memorial (SSKM) Hospital, Kolkata, IND

**Keywords:** bone formation, chondroid syringoma, eyelids, skin adnexa, tumor

## Abstract

A chondroid syringoma is a rare primary benign skin adnexal tumor with distinct histomorphology. However, extensive metaplastic bone formation within the tumor can raise diagnostic concerns. Further, the eyelid is quite a rare site of involvement of this tumor. Herein, we report a case of a chondroid syringoma with metaplastic bone formation involving the right upper eyelid in a 60-year-old female patient. The Ki67 labeling index was low. On follow-up for 36 months, no recurrence was noted. Acquaintance with the diverse histomorphological and clinical presentation of this tumor can prevent major diagnostic pitfalls.

## Introduction

A chondroid syringoma is a rare benign primary skin adnexal tumor with distinct histomorphology. The tumor is rare with an incidence of 0.01-0.098% [1]. It typically affects the middle-aged population and has a male preponderance [2]. The typical histomorphology includes a multilobulated biphasic tumor centered in the dermis, composed of both epithelial and stromal components. The epithelial component forms ducts, glands, and cysts, while the stromal component shows a prominent chondromyxoid change. The histomorphological divergence with metaplastic adipocytic, osseous, or fibrous elements in the stroma makes diagnosis challenging. Extensive osseous metaplasia has limited case reports and hence necessitates exposition. Further, the tumor has a predilection for the head and neck region. The eyelid is a rare site of involvement with chondroid syringomas accounting for 0.48% of all eyelid tumors [3]. Osseous metaplasia in a chondroid syringoma is rare with extensive bone formation extremely rare [4].

Herein we report a case of a chondroid syringoma with profound osseous metaplasia in a 60-year-old female patient.

## Case presentation

**Figure 1:**
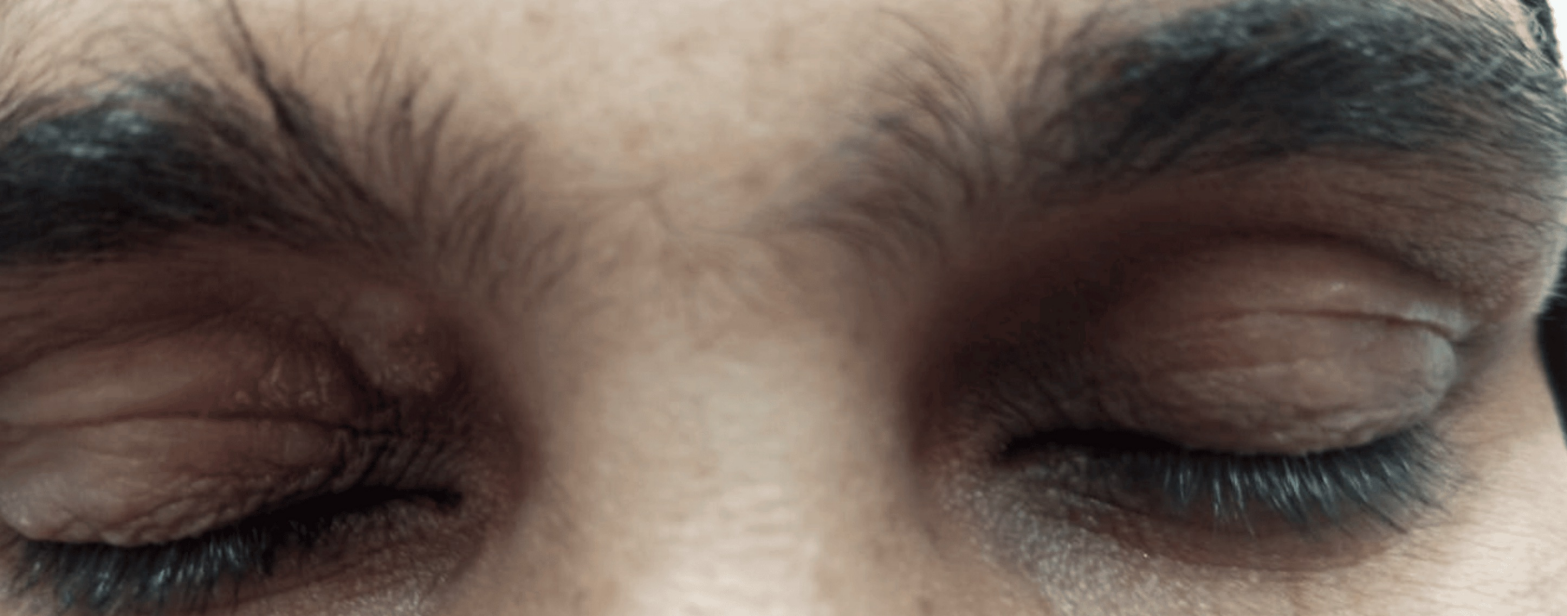
Clinical picture of a right upper eyelid swelling measuring 1.5 x 1 cm

A 60-year-old female patient presented with a nodule measuring 1.5 x 1 cm, above the right upper eyelid (Figure 1). Clinical history revealed that this swelling had been slowly growing for the past three years. A clinical diagnosis of basal cell carcinoma (BCC) was made. A Tru-cut biopsy was performed, and a microscopic diagnosis of a chondroid syringoma was made.

**Figure 2:**
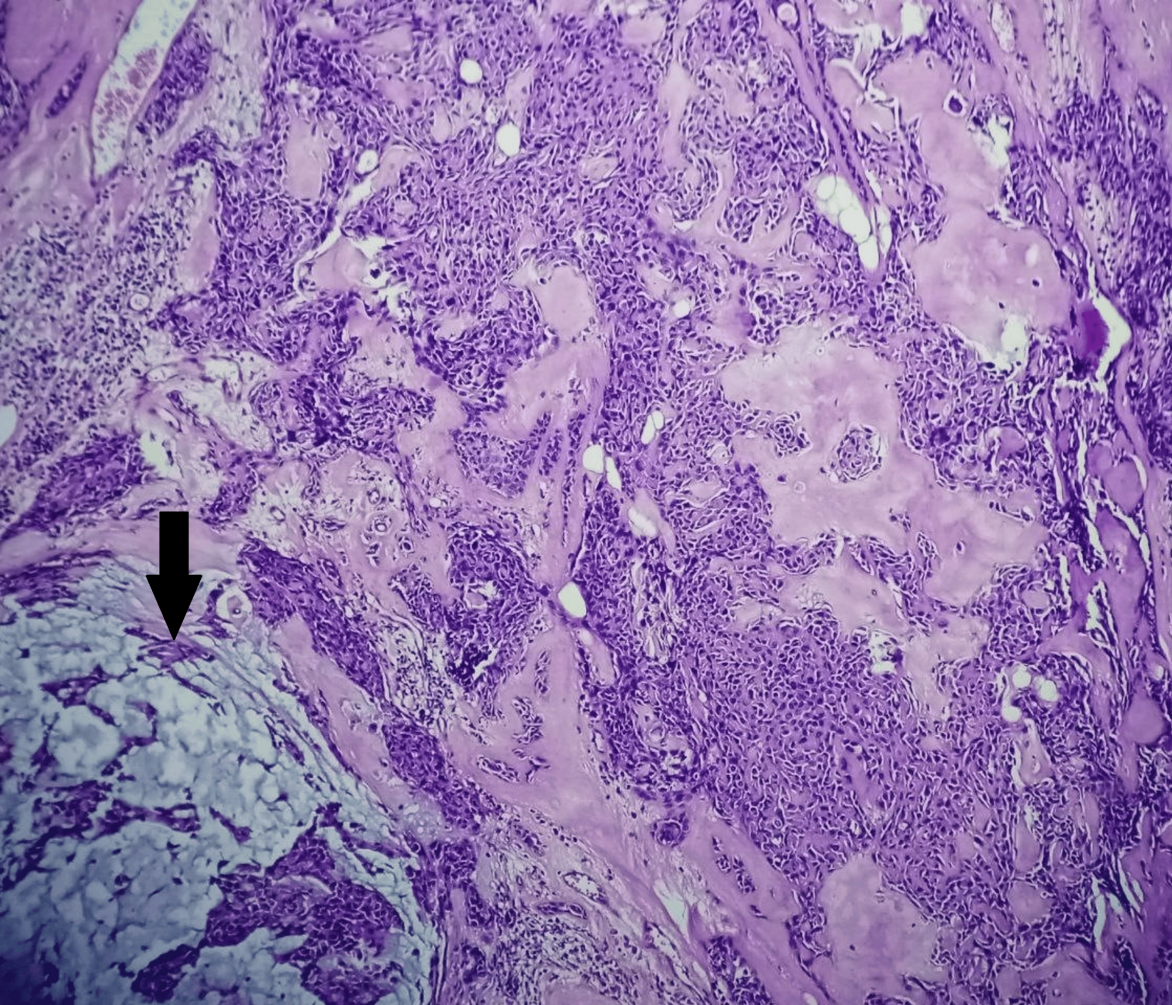
Photomicrograph showing nests, cords, tubules, and sheets of round-to-ovoid tumor cells. Extensive areas of metaplastic bone formation are noted. The surrounding stroma was variably chondroid with muco-myxoid areas, marked with a black arrow (H&E, 100X)

**Figure 3:**
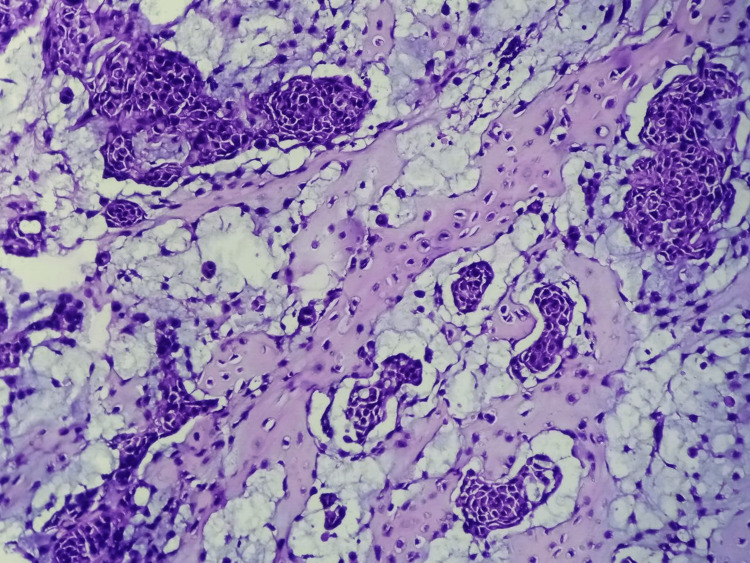
Photomicrograph showing nests of tumor cells in a muco-myxoid stroma with extensive areas of metaplastic bone formation (H&E, 400X)

**Figure 4:**
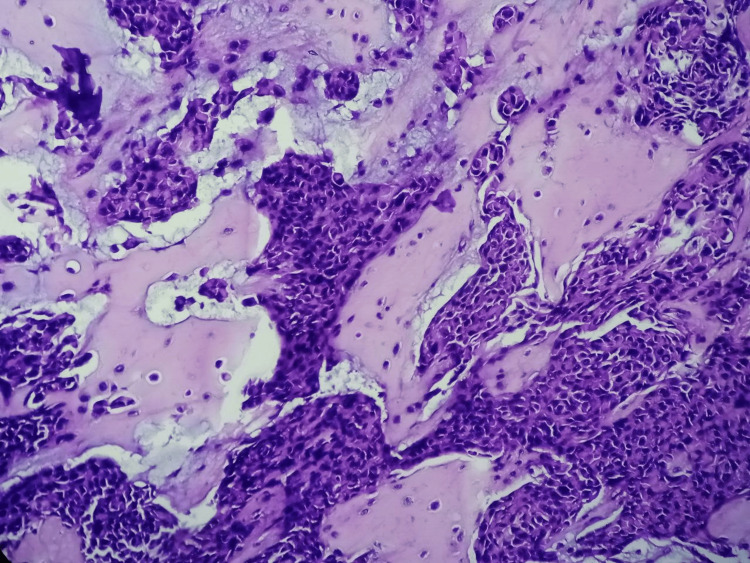
Photomicrograph showing nests of tumor cells with extensive areas of metaplastic bone formation (H&E, 400X)

The lesion was surgically excised and was sent for histopathological examination. After overnight fixation in buffered 10% neutral buffered formalin, the specimen was grossed, and formalin-fixed paraffin-embedded (FFPE) blocks were prepared. Subsequently, 5 μm sections were cut and stained with routine hematoxylin-eosin (H&E) stain. On microscopic examination, a tumor was seen to be composed of nests, cords, tubules, and sheets of round-to-ovoid tumor cells with finely dispersed nuclear chromatin and inconspicuous nucleoli. Mitotic activity was sparse. There was no atypical mitosis, necrosis, or infiltrative margins. However, extensive areas of metaplastic bone formation were found. The surrounding stroma was variably chondroid with muco-myxoid areas. Immunohistochemistry (IHC) was not required to establish a diagnosis, as the morphology was obvious. However, IHC for Ki67 was done to ascertain the proliferative potential of the tumor. Ki67 labeling index was <1%. Hence, a diagnosis of a chondroid syringoma with metaplastic bone formation was made (Figures 2-4). On follow-up for a period of 36 months, no recurrence was observed.

## Discussion

Billroth, in 1859, described a chondroid syringoma for the first time as a type of salivary gland tumor composed of both cartilaginous and muco-myxoid areas [5]. In 1961, Hirsch and Helwig were the first to introduce the term ‘chondroid syringoma’, while describing a mixed tumor of the skin originating from the eccrine glands [6]. Histologically, the tumor has a distinct histomorphology with both epithelial and stromal components. This tumor is also the analog of the pleomorphic adenoma of the salivary gland. Chondroid, adipocytic, and rarely osseous metaplasia have been reported in this entity. Of these, osseous metaplasia is extremely rare and makes diagnosis challenging, especially when excluding bone invasion in malignant chondroid syringoma. Further, this entity frequently affects the scalp, forehead, nose, cheek, chin, and upper lip. Rarely, the external auditory canal, eyelid, and scrotum have been involved [7]. The eyelid is quite a rare site with under 30 cases reported worldwide [3]. 

To date, to the best of our knowledge, only six cases [4,8-12] have been reported showing osseous metaplasia. Histologically, the tumor is centered in the deeper dermis and subcutaneous fat and shows a multilobulated architecture. Besides having a chondroid and myxoid stroma, the tumor also shows epithelial and myoepithelial elements arranged in glands, nests, and cysts. Stroma is periodic acid-Schiff (PAS) and Alcian blue (AB) positive. In addition to the above features, our case also revealed large islands of metaplastic bone formation. Osseous metaplasia, though reported previously, is often focal, unlike in our case, which showed considerable bone formation. This was similar to that reported by Bedir et al. [4]. The pathogenesis behind the bone formation is still unknown but could be attributed to the endochondral ossification similar to that observed in growing bones [8]. However, Shimizu et al. suggested that bone formation is without any intervening cartilage and may be a result of frank deposition of osteoid by the metaplastic cells [9]. Further, a few authors have suggested that pluripotent stem cells could be a cause of this phenomenon and noted the presence of ossification together with hair matrix, and sebaceous duct differentiation [10,11]. Furthermore, studies have suggested that modified myoepithelial cells can also lay down bone [13]. Additionally, Awasthi et al. reported extensive ossification with marrow formation [12].

Chondroid syringomas are benign subcutaneous tumors with well-described histomorphology. The size of the tumor is generally 0.5-3 cm; however, sizes larger than 10 cm have also been reported. Malignant chondroid syringomas are rare and are usually greater than 3 cm in size [14]. Here, in our case, the tumor was around 1.5 cm in size, and it did not show any features of malignancy.

Chondroid syringomas have several mimickers both clinically and histologically. Clinically, the tumor mimics a melanocytic nevus, pyogenic granuloma, basal cell carcinoma, dermatofibroma, dermoid cyst, and other skin adnexal tumors [15]. Histopathologically, the tumor often overlaps with cutaneous chondromas, chordomas, myoepitheliomas, and malignant chondroid syringomas [16-19]. Cutaneous chondromas are benign cartilaginous tumors predominantly involving the distal extremities. Histologically, the tumor shows lobules of hyaline cartilage but lacks epithelial and myoepithelial components [16]. Further, a chordoma is characteristically a midline malignant tumor of notochordal derivation. Histologically, it shows a lobular architecture with the extracellular myxoid stroma and typical physaliphorous cells. However, a rare case of cutaneous metastasis of a chondroid chordoma has been described by Riesco-Martínez et al. [17], which can pose a diagnostic challenge. IHC using brachyury can often aid in the diagnosis of such cases. A chondroid syringoma is classified as a myoepithelial tumor of the skin and soft tissue and has a close resemblance to the pleomorphic adenoma of the salivary gland [18]. Myoepitheliomas, histologically, closely mimic chondroid syringomas and are characterized by a lobular growth pattern with tumor cells having clear cell, plasmacytoid, or spindled morphology and a hyaline to myxoid to chondroid stroma. However, about 45% of myoepitheliomas and myoepithelial carcinomas possess an alteration in the EWSR1 gene unlike PLAG 1 gene alteration noted in chondroid syringoma [18]. 

Furthermore, malignant chondroid syringomas show an infiltrative growth pattern, a higher degree of nuclear pleomorphism, and necrosis [19]. Additionally, Nel et al. in a report of two cases of malignant chondroid syringomas found that both the epithelial and stromal components can be malignant with mesenchymal stromal component revealing sarcomatous differentiation [20]. 

## Conclusions

A chondroid syringoma is a rare primary benign skin adnexal tumor with distinct histology. However, extensive metaplastic bone formation within this tumor raises a concern for a histopathologist. Further, the eyelid is quite a rare site of involvement. Hence, acquaintance with the diverse histomorphological and clinical presentation of this tumor can prevent diagnostic pitfalls.

## References

[1] Yavuzer R, Başterzi Y, Sari A, Bir F, Sezer C (2003). Chondroid syringoma: a diagnosis more frequent than expected. Dermatol Surg.

[2] Kakuta M, Tsuboi R, Yamazaki M, Sakuma M, Yoshikata R, Ogawa H (1996). Giant mixed tumor of the face. J Dermatol.

[3] Kumar MA, Srikanth K, Vathsalya R (2013). Chondroid syringoma: a rare lid tumor. Indian J Ophthalmol.

[4] Bedir R, Yurdakul C, Sehitoglu I, Gucer H, Tunc S (2014). Chondroid syringoma with extensive bone formation: a case report and review of the literature. J Clin Diagn Res.

[5] Sirivella S, Gielchinsky I (2010). Chondroid syringoma: a rare tumor of the chest wall. Ann Thorac Surg.

[6] Hirsch P, Helwig EB (1961). Chondroid syringoma. Mixed tumor of skin, salivary gland type. Arch Dermatol.

[7] Kaushik V, Bhalla RK, Nicholson C, de Carpentier JP (2005). The chondroid syringoma: report of a case arising from the external auditory canal. Eur Arch Otorhinolaryngol.

[8] Eccher A, Brunelli M, Gobbo S (2007). Chondroid syringoma with extensive ossification. Int J Surg Pathol.

[9] Shimizu S, Han-Yaku H, Fukushima S, Shimizu H (1996). Immunohistochemical study of mixed tumor of the skin with marked ossification. Dermatology.

[10] Paul K, Sreekar H, Dhanraj P, Lamba S, George SM (2011). Chondroid syringoma with extensive ossification. Ann Maxillofac Surg.

[11] Akasaka T, Onodera H, Matsuta M (1997). Cutaneous mixed tumor containing ossification, hair matrix, and sebaceous ductal differentiation. J Dermatol.

[12] Awasthi R, Harmse D, Courtney D, Lyons CB (2004). Benign mixed tumour of the skin with extensive ossification and marrow formation: a case report. J Clin Pathol.

[13] Mandal S, Dhingra K, Roy S, Khurana N (2008). Extensive bone with marrow formation in pleomorphic adenoma. Report of a case. ANZ J Surg.

[14] Hudson LE, Craven CM, Wojno TH, Grossniklaus HE (2017). Giant chondroid syringoma of the lower eyelid. Ophthalmic Plast Reconstr Surg.

[15] Huang QF, Shao Y, Yu B, Hu XP (2022). Chondroid syringoma of the lower back simulating lipoma: a case report. World J Clin Cases.

[16] Thompson J, Squires S, Machan M, Fraga GR, Aires D (2012). Cutaneous mixed tumor with extensive chondroid metaplasia: a potential mimic of cutaneous chondroma. Dermatol Online J.

[17] Riesco-Martínez MC, Parrilla-Rubio L, Enguita-Valls AB, Delgado-Márquez AM, Ruste SA, López-Martín JA (2016). A unique case of distant skin metastasis from chondroid chordoma. JAAD Case Rep.

[18] Jo VY, Fletcher CD (2015). Myoepithelial neoplasms of soft tissue: an updated review of the clinicopathologic, immunophenotypic, and genetic features. Head Neck Pathol.

[19] Malik R, Saxena A, Kamath N (2013). A rare case of malignant chondroid syringoma of scalp. Indian Dermatol Online J.

[20] Nel CE, van der Byl D, Grayson W (2019). Malignant chondroid syringoma: a report of two cases with a sarcomatous mesenchymal component. Dermatopathology (Basel).

